# Waves of visibility: probing the depth of inter-ocular suppression with transient and sustained targets

**DOI:** 10.3389/fpsyg.2014.00804

**Published:** 2014-07-30

**Authors:** Lisandro N. Kaunitz, Alessio Fracasso, Māris Skujevskis, David Melcher

**Affiliations:** ^1^School of Psychological Sciences, Department of Medicine, Nursing and Health Sciences, Monash UniversityMelbourne, VIC, Australia; ^2^Experimental Psychology, Helmholtz Institute, Utrecht UniversityUtrecht, Netherlands; ^3^Cognitive Neuroscience Sector, International School for Advanced StudiesTrieste, Italy; ^4^Center for Mind/Brain Sciences (CIMeC), University of TrentoTrento, Italy; ^5^Department of Cognitive Sciences, University of TrentoTrento, Italy

**Keywords:** visual awareness, metacognition, continuous flash suppression, masking, vision, binocular, consciousness

## Abstract

In order to study non-conscious visual processing, researchers render otherwise consciously perceived images into invisible stimuli. Through the years, several psychophysical techniques have been developed for this purpose. Yet the comparison of experimental results across techniques remains a difficult task as the depth of suppression depends on the interactions between the type of stimuli and the suppression methods employed. This poses a limit to the inferences that researchers make about the extent of non-conscious processes. We investigated the mechanisms underlying inter-ocular suppression during continuous flash suppression (CFS) and dichoptic visual masking using a transient onset target stimulus and a variety of stimulus/mask temporal manipulations. We show that target duration, timing of target onset, and mask frequency are key aspects of inter-ocular suppression during CFS with transient targets. The differences between our results and sustained target CFS studies suggest that two distinct mechanisms are involved in the detection of transient and prolonged target stimuli during CFS. Our results provide insight into the dynamics of CFS together with evidence for similarities between transient target CFS and dichoptic visual masking.

## Introduction

The notion that we lack conscious access to most of our brain activity is not new. For decades, researchers have inferred visual non-conscious processes in the brain from subjects' responses to invisible stimuli. What kind of information can the visual system encode without consciousness? The answer to this question depends on the definition of consciousness adopted by researchers and the interaction between the kind of stimuli (e.g., simple lines, images of faces or motion stimuli) and the psychophysical techniques (e.g., inter-ocular suppression, visual crowding, or backward masking) that render those stimuli perceptually invisible (Faivre et al., 2014; Izatt et al., 2014). Different behavioral paradigms achieve stimulus invisibility in different ways and at different levels of neural processing, making the comparison of results among different methods difficult (Kim and Blake, 2005; Fogelson et al., 2014).

Several psychophysical techniques make stimuli invisible via inter-ocular suppression: dissimilar images are presented to the left and right eyes, which leads to the suppression of one of the images from conscious perception. For example, in binocular rivalry (Wheatstone, 1838; Blake and Fox, 1974) two different stimuli are presented each to one eye making the subject's conscious perception alternate between the two. In dichoptic masking (Schiller and Smith, 1968), a mask is shortly presented to one eye immediately before or after a brief target is presented to the other eye. In flash suppression (Wolfe, 1984) a stimulus is initially presented to one eye and after several milliseconds a dissimilar stimulus is presented to the other eye, suppressing the first stimulus for several hundred milliseconds (flash suppression differs from dichoptic masking in that the two stimuli temporally overlap after the onset of the second stimuli). Among these techniques continuous flash suppression (CFS; Tsuchiya and Koch, 2005) constitutes the strongest version, capable of masking stimuli for prolonged periods of time (several seconds). It achieves long periods of suppression with a train of mask patterns (usually referred to as “Mondrians”) flashed in rapid succession to one eye while a (typically static) target stimulus is presented to the other eye. CFS has proved a suitable tool for investigating the effects of non-conscious stimuli on, for example, face adaptation (Alais and Melcher, 2007; Stein and Sterzer, 2011), afterimage formation (van Boxtel et al., 2010), and motion processing (Kaunitz et al., 2011, 2013 for a comprehensive review on the scope and limits of non-conscious processing see Lin and He, 2009).

Currently, it is not clear what common mechanisms regulate the balance between visibility and suppression under the various scenarios of dichoptic stimulation. It has been proposed that transient stimuli (of a few tens of milliseconds) under dichoptic visual masking are detected through a “transient channel” triggered by the spatiotemporal edges of the stimulus (Macknik et al., 2000; Breitmeyer and Öğmen, 2006). Target suppression is strongest just before and immediately after a stimulus onset asynchrony (SOA) of 0 ms, suggesting a close relationship between the mask-to-target temporal distance and stimulus visibility. For prolonged presentation of stimuli, it has been reported that CFS exerts its strongest suppression with Mondrians at a frequency of ~10 Hz (Tsuchiya et al., 2006) but with the exception of this pioneering study, the influence of Mondrian frequency on stimulus suppression has not been investigated systematically.

We conducted four experiments to investigate the dynamics of CFS and the mechanisms of suppression for transient (starting from a few milliseconds) and prolonged (up to several hundred milliseconds) stimuli. The manipulation of the relative temporal distance of presentation between targets and Mondrians allowed us to study the temporal dynamics of stimuli suppression during CFS. Moreover, we investigated the effect of Mondrian frequency and target duration on objective performance and subjective reports of target visibility. We aimed to clarify how the temporal dynamics of CFS determine the depth of suppression for both transient and prolonged stimuli and, in particular, how the timing of the presentation of stimuli within the sequence of Mondrians would affect objective performance, subjective visibility and metacognition—an objective unbiased measure of subjects' awareness of their own responses.

## Materials and methods

### Subjects

Three authors and 29 naïve subjects (mean age: 28.4 years, range: 23–41 years) participated in the experiments. They all had normal or corrected-to-normal visual acuity. Prior to the experiments all participants gave their informed consent according to the guidelines of the University of Trento ethical committee.

### Apparatus

Stimuli were generated on a PC running Matlab and Psychtoolbox 3.0 (Brainard, 1997; Pelli, 1997). They were presented on a 21″ Phillips Brilliance 109P4 CRT monitor with a refresh rate of 85 Hz and a screen resolution of 1280 × 1024 pixels. Except for the luminance of the monitor, the room was otherwise dark. Stimuli were viewed through a mirror stereoscope and a headrest was used to maintain a constant viewing distance of 60 cm.

### Stimuli

**Figure 1:**
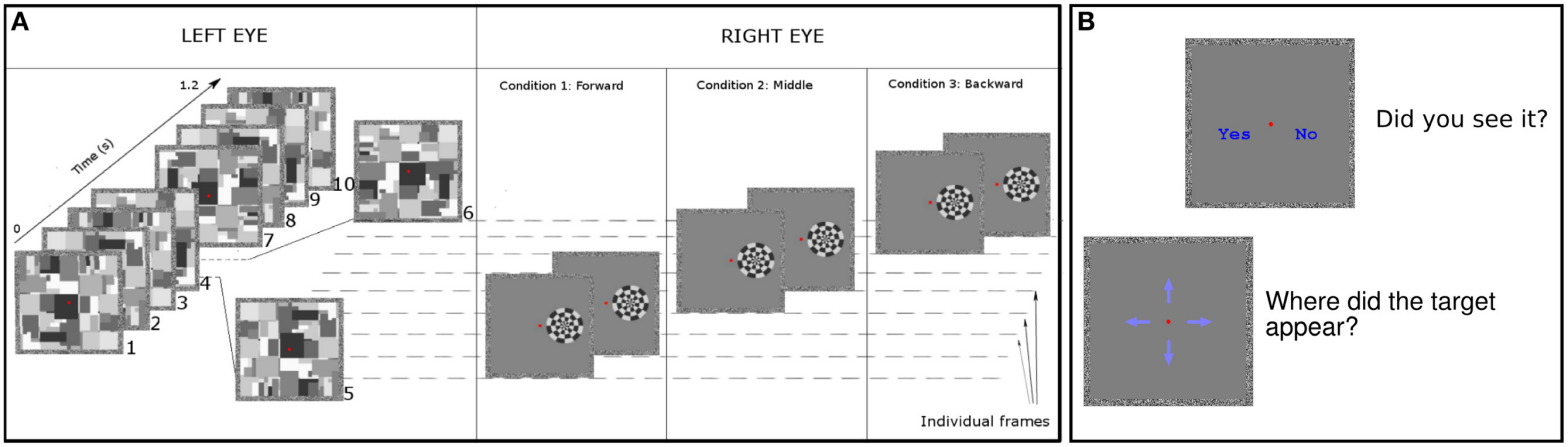
**Experimental design. (A)** We presented Mondrian patterns to left eye of subjects (leftmost panel in the figure) at frequency of 8.5 Hz in Experiment 1. For Experiment 2 we used a range of frequencies. Targets were always displayed to the right eye of subjects. As an example, we show three trials occurring between the 5th and 6th Mondrian presentation. *Forward condition*: we displayed targets on the screen in the following two frames (~24 ms) after the Mondrian. *Middle condition*: we presented targets at an equal temporal distance between the previous and subsequent Mondrian. *Backward condition*: we displayed targets for two frames before displaying the Mondrian. In all conditions the Mondrian's luminance polarity changed from the first frame to the second frame (see Materials and Methods Section). **(B)** On each trial, after the stimulus presentation we asked subjects to (1) report the location of the target on the screen and (2) to indicate whether they had perceived the target among the Mondrian sequence.

Two square frames (8° visual angle) were presented on the display to help stabilize binocular fusion. Before subjects started the experiments the mirror stereoscope was manually adjusted until subjects achieved binocular fusion of the two frames and reported seeing only one single frame in their visual field. Throughout the experiments we used a circular black and white checkerboard as a target and a series of Mondrian masks to create CFS. The checkerboard target was 3 × 3° of visual angle in size and was formed by 12 equally sized sectors, each of which was further subdivided into four sections along the radius. The checkerboard was always presented at an eccentricity of 2° from the fixation point against a gray background (CIE coordinates: *x* = 0.29, *y* = 0.32, *z* = 0.39; luminance = 18.2 cd/m^2^). Mondrians were created by randomly superimposing black, white and gray squares. Mondrians subtended 8° of visual angle and filled the entire inner area of the external frames (Figure 1). Prior to each experimental session 40 Mondrians were created and 10 of these were randomly selected for each trial. Checkerboards were presented to the right eye while Mondrians were always presented to the left eye.

### Procedures

In all experiments the subjects' task was to detect and report the location of the checkerboard target. Targets were presented for 24 ms (two video frames) in Experiments 1–3, and with variable durations during Experiment 4. Checkerboards changed luminance polarity (white areas turning black and black areas turning white) on their second frame of presentation (Figure 1). Each trial lasted 1.5 s, during which Mondrians were presented at their corresponding frequency (see below).

#### Objective detection

At the end of each trial four blue arrows appeared on the screen and participants had to indicate the location where the checkerboard had been presented on the screen. They responded “up,” “down,” “left” or “right” in a 4 alternative forced choice (AFC) using the four arrow keys of the keyboard (Figure 1B).

#### Subjective visibility

Immediately after the 4AFC subjects were presented with two options: “yes” or “no” to inquire whether or not they had perceived the target. This 2AFC provided a trial-by-trial subjective measure of target visibility. To avoid expectation biases we randomly varied the inter-trial intervals among the values of 250, 500, 750, or 1000 ms. Prior to the main experiment subjects performed two training block (one in Experiment 4). Results from the training blocks were excluded from data analysis.

### Experiment 1. the time course of masking with CFS

Twelve subjects (three authors) participated in Experiment 1. Mondrians were presented at a frequency of 8.5 Hz. Checkerboards were presented within a time window of 500–900 ms after trial onset. At least 5 Mondrians were presented on the screen before the appearance of the checkerboard. Targets appeared after the 5th, 6th, 7th, or 8th Mondrian. Checkerboards were presented at 4, 12, 16, 24, and 64% of Michelson contrast. To study the effect that temporal distance between checkerboard and Mondrian presentation had on target detection we presented three different timing conditions (Figure 1). In the ‘backward’ condition checkerboards were presented on the two video frames immediately preceding the presentation of a new Mondrian. In the “middle” condition, the target was presented on the two video frames temporally situated in the middle between two Mondrians. In the “forward” condition, targets were presented on the two video frames immediately following the presentation of a Mondrian.

In addition to the three timing conditions, we included a condition in which targets were always presented between the 2nd and 3rd Mondrian, mimicking the “middle” condition but at the beginning of the train of Mondrians. This condition allowed us to investigate the role of the initial train of Mondrians, since in previous studies it was hypothesized that suppression might build up in strength with repeated flashing of the Mondrians (CFS) compared to only one or a few flashes (Tsuchiya et al., 2006). Each subject performed 64 trials for each contrast and timing condition in 6 counterbalanced blocks of 160 trials amounting to a total of 960 trials (~1 h of experiment). Sixteen checkerboards were presented in each location for each contrast × timing condition.

### Experiment 2. the effects of mask frequency on target suppression

Twelve subjects (three authors) participated in Experiment 2. The main goal of this experiment was to assess the effect of mask frequency on the detection of brief targets. For this reason we presented the Mondrians at five temporal frequencies: 5.3, 8.5, 10.6, 16.6, and 28.5 Hz. The checkerboards were presented at 12 and 16% of Michelson contrast. We presented the checkerboards only in the backward condition (Figure 1). Targets appeared after the 5th, 6th, 7th, or 8th Mondrian. Each subject performed 40 trials for each contrast x Mondrian frequency, in 5 counterbalanced blocks of 80 trials amounting to a total of 400 trials (~35 min of experiment). Each frequency was tested in a block and the order of presentation of blocks was randomized across subjects to avoid learning effects. A total of 10 checkerboards were presented in each location for each contrast × timing condition.

### Experiment 3. comparing CFS and inter-ocular “sandwich” masking

Twelve subjects (one author) participated in this experiment. We aimed to evaluate the depth of suppression induced by CFS as compared to a brief inter-ocularly presented “sandwich” (forward plus backward) mask for the detection of briefly presented targets. All contrast and Mondrian frequency parameters were identical to Experiment 2, except that only two Mondrians (one preceding and one following the target) were presented on each trial. Apart from the differences in the number of Mondrian masks presented, the other parameters, i.e., the number of trials, blocks, conditions, and total duration of the experiment were identical to Experiment 2.

### Experiment 4. brief vs. prolonged target presentation

Twelve subjects participated in this experiment. One of the subjects had to be discarded from further analysis as he claimed to be unable to detect/see any targets during the experiment. The main goal of this experiment was to assess the effect of target duration on the detection of targets. Targets were presented for 24, 48, 70, 118, and 506 ms. They changed polarity only once after the first video frame. We presented Mondrians at three temporal frequencies: 8.5, 16.6, and 28.5 Hz. The checkerboards were presented at 12% of Michelson contrast and they appeared after the 5th, 6th, 7th, or 8th Mondrian. Each subject performed 40 trials for each target duration × Mondrian frequency, in 10 counterbalanced blocks of 60 trials amounting to a total of 600 trials (~45 min of experiment). All conditions were presented intermixed in each block. A total of 10 checkerboards were presented in each location for each target duration × Mondrian frequency condition.

### Analyses

In all four experiments we assessed subjects' objective performance, subjective visibility reports and metacognition. We calculated *objective performance* as the proportion of correct responses in the 4AFC and we assessed *subjective visibility* as the proportion of “seen” trials in the 2AFC. By metacognition we mean the ability of subjects to discriminate between their own correct and incorrect responses when they claim to see the targets. In order to obtain a measure of metacognition we used the subjects' binary confidence ratings of their own responses (i.e., the “yes, target seen”/“no, target not seen” responses). First, we divided subjects' responses into two groups: correct and incorrect responses. Second, we calculated the proportion of correct trials “seen” and of incorrect trials “seen.” We considered “seen” responses as analogous to “high confidence” responses in previous studies (Kunimoto et al., 2001). Third, we calculated hit rates as the proportion of seen and correct trials over all correct responses, and false alarm rates as the proportion of seen and incorrect responses over all incorrect responses. From these hit and false alarm rates we calculated d primes for each subject. This measure of metacognition is known as the type II d prime (Kunimoto et al., 2001).

## Results

### Experiment 1

**Figure 2:**
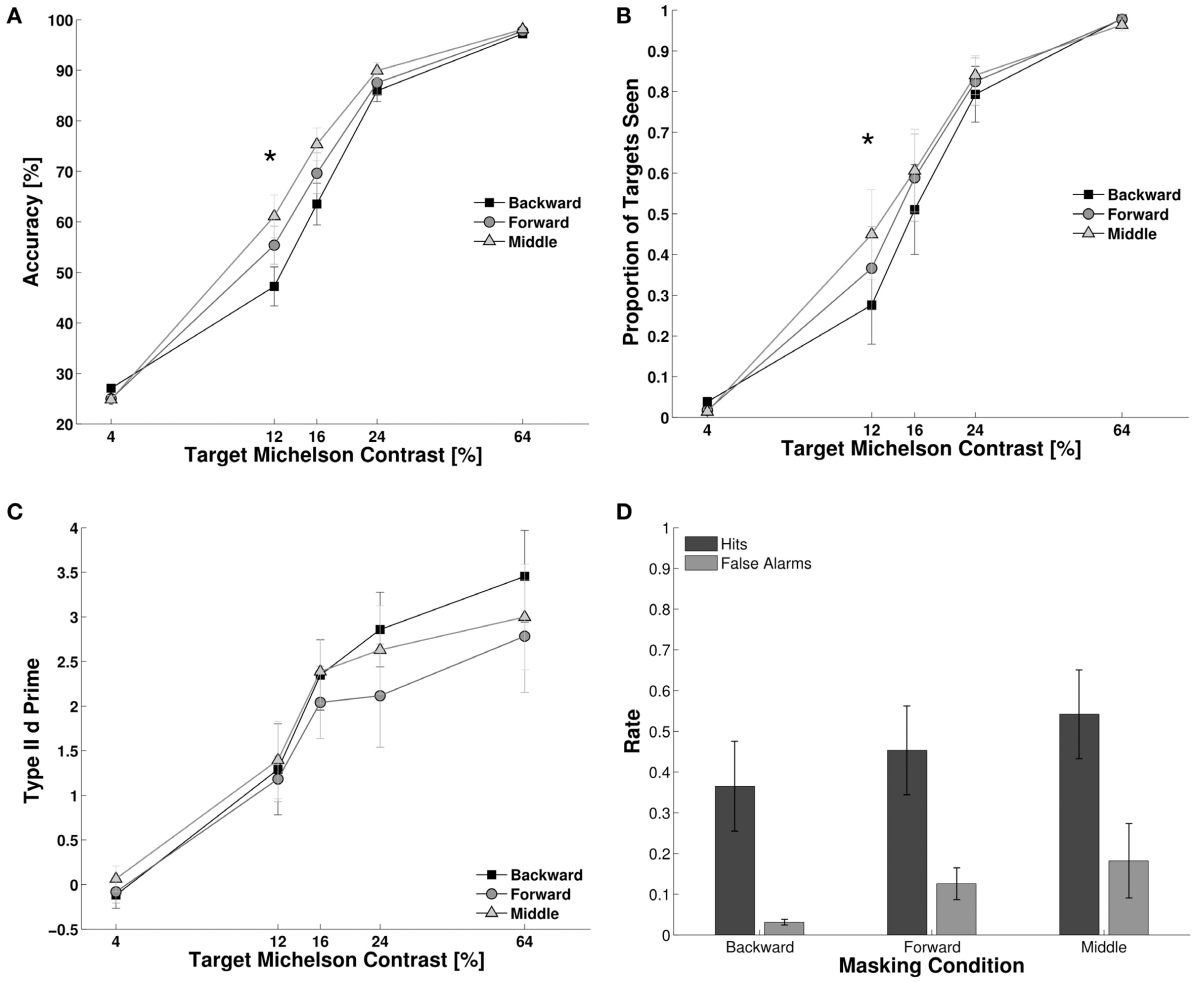
**Experiment 1**. We studied the effect of our experimental conditions on target suppression. **(A–C)** Lower performance indicates higher suppression. **(A)** Objective performance for target detection. The effect of suppression was strongest for the backward masking condition. Subjects were more accurate in the middle condition than in the backward masking conditions for the 12 and 16% contrast levels. **(B)** Target visibility showed a similar trend as to objective performance, subjects reported to see more targets in the middle condition than in the backward condition. **(C)** Metacognition was equivalent for the three masking conditions at all contrast levels (see statistics in the Results Section). **(D)** Hit and false alarm rates for trials where subjects reported to consciously see the target. The panel shows the 3 masking conditions at 12% contrast level. The linear increase in the proportion of targets that subjects reported as “seen” across masking conditions was correlated with an increase in hits rate, but also with an increase in the false alarm rates. This resulted in an equivalent metacognition across masking conditions. *N* = 12, error bars represent one s.e.m. Asterisks indicate *p* < 0.05 (corrected for multiple comparisons, see Results Section).

We studied the effects of the three timing conditions within CFS (backward, forward, and middle, see Materials and Methods) on objective accuracy, subjective reports and metacognition (Figure 2A). First, we assessed subjects' objective performance for target detection employing Two-Way repeated measures ANOVA, with timing condition and contrast as factors. We observed main effects for contrast, *F*_(4, 44)_ = 56.01, *p* < 0.001, partial eta-squared (*N_P_*^2^) = 0.83, and timing condition, *F*_(2, 22)_ = 17.60, *p* < 0.001, *N_P_*^2^ = 0.61, as well as a significant interaction parameter, *F*_(8, 88)_ = 4.17, *p* = 0.002. *Post-hoc* Bonferroni corrected paired *t*-tests showed a statistical difference between backward and middle masking conditions at 12 and 16% contrast levels (*t* = −3.22, *df* = 11, *p* = 0.008; and *t* = −3.61, *df* = 11, *p* = 0.004, respectively). Second, we measured subjective visibility by counting the proportion of targets reported as seen in the 2AFC. We observed a main effect for contrast, *F*_(4, 44)_ = 52.70, *p* < 0.001, for timing condition, *F*_(2, 22)_ = 11.6, *p* < 0.001, *N_P_*^2^ = 0.82, and a significant interaction parameter *F*_(8, 88)_ = 5.7, *p* < 0.001, *N_P_*^2^ = 0.34. We did not observe any statistical difference among timing conditions (Figure 2B, *post-hoc* Bonferroni corrected paired *t*-tests). Finally, we assessed subjects' metacognition (Figure 2C). We observed a main effect for contrast, *F*_(4, 44)_ = 23.80, *p* < 0.001, *N_P_*^2^ = 0.68, on type II d primes, but no effect for the timing conditions, *F*_(2, 22)_ = 1.50, *p* = 0.247, nor for the interaction parameter, *F*_(8, 88)_ = 0.26, *p* = 0.960. To directly compare objective performance, subjective visibility and metacognition we computed the z-score of each dependent variable across all conditions and participants. Using this normalized measure we computed the difference between “middle masking condition” and “backward masking condition” for each participant and dependent variable. Significance was assessed by a within participant ANOVA, with variable type as the independent variable, *F*_(2, 22)_ = 9.103, *p* = 0.001, *N_P_*^2^ = 0.44. Bonferroni corrected *post-hoc* analysis revealed a difference between subjective visibility and metacognition, *t*_(11)_ = 3.09, *p* = 0.02, and between objective performance and metacognition, *t*_(11)_ = 3.41, *p* = 0.01 (see Supplementary Figure 1).

The pattern of data showed that masking types affected objective performance and visibility reports at 12 and 16% of contrast levels. However, metacognition did not vary with masking condition (Figure 2D). This suggests that the ability of subjects to discriminate between their own correct and incorrect responses remained equal across masking conditions in spite of the increases in objective performance for the middle condition. Even though subjects were more accurate and reported seeing more targets in the middle than in the backward conditions at 12 and 16% of contrast levels, the hit and false alarm rates increased proportionally when subjects claimed to see the target. For the 12% contrast level we observed a main effect of masking condition, *F*_(2, 22)_ = 12.10, *p* < 0.001, *N_P_*^2^ = 0.52, a main effect of response type [hit rate vs. false alarm rate, *F*_(1, 11)_ = 16.9, *p* < 0.001, *N_P_*^2^ = 0.60] but no significant interaction parameter, *F*_(2, 22)_ = 0.06, *p* = 0.87. The linear increase in hits and false alarms results in identical type II d primes across conditions, which indicates that subjects had a better objective performance at detecting targets without an increase in their metacognition, i.e., without being more accurate in their judgments about their own correct and incorrect responses.

### Experiment 2

**Figure 3:**
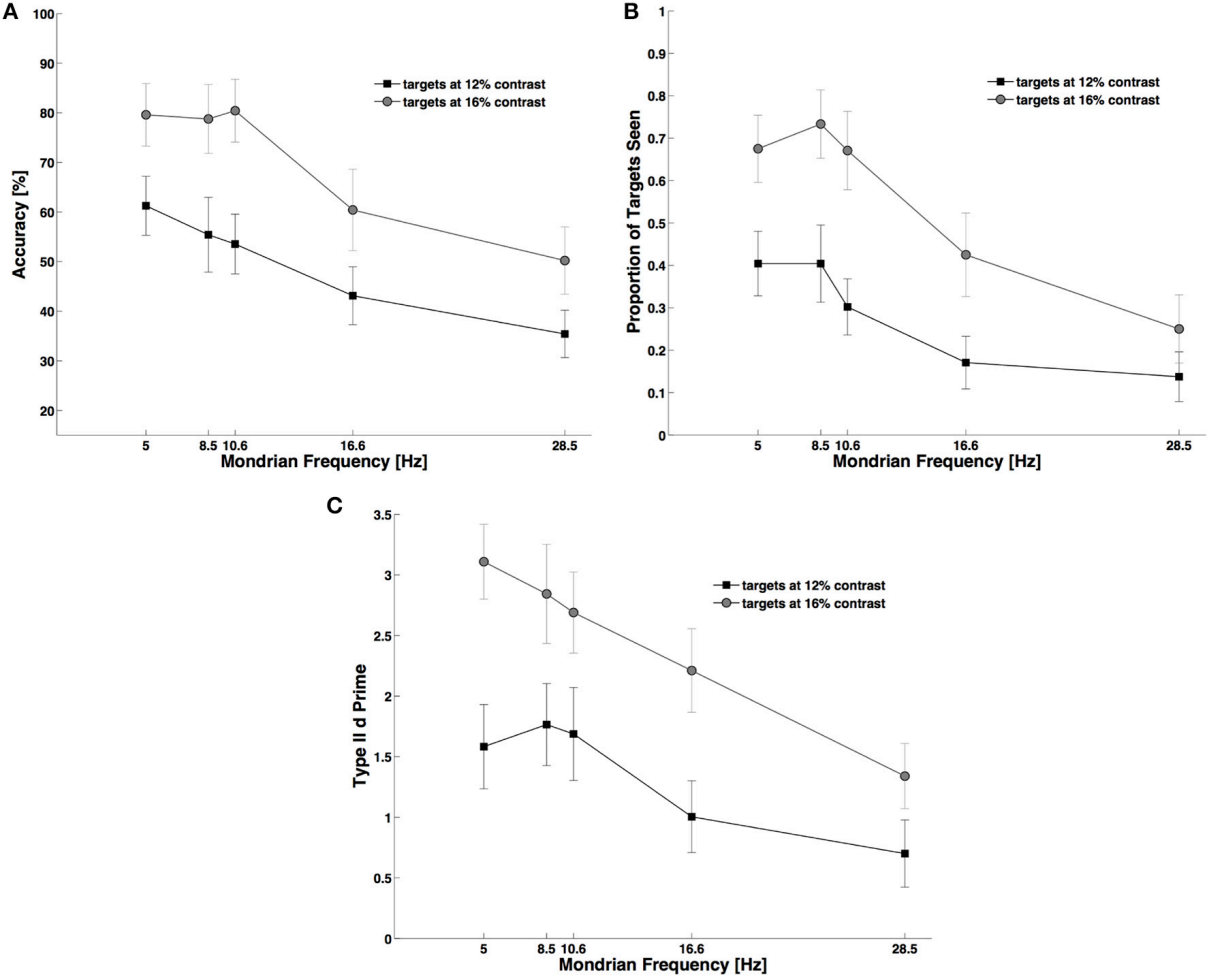
**Experiment 2**. During CFS higher Mondrian frequencies generated stronger suppression of targets and a decrease in subjects' performance. The same decaying trend equally affected: objective performance **(A)**, subjective reports of visibility **(B)**, and metacognition **(C)**. For this experiment we used only trials in the backward condition (see Figure 1 and Materials and Methods Section). As with Figure 1, in (**A–C**) lower performance indicates higher suppression from the Mondrian masks.

As expected, the frequency of presentation of Mondrians affected all three measures, but we found that performance generally decreased as a function of frequency. For objective performance at detecting the target (Figure 3A) we observed a main effect of frequency, *F*_(4, 44)_ = 12.50, *p* < 0.001, *N_P_*^2^ = 0.53, and a main effect of contrast, *F*_(1, 11)_ = 76.80, *p* < 0.001, *N_P_*^2^ = 0.87, with no interaction between these main factors, [*F*_(4, 44)_ = 1.46, *p* < 0.232]. Subjective reports of visibility (Figure 3B) also decreased with decreasing contrast, *F*_(1, 11)_ = 46.60, *p* < 0.001, *N_P_*^2^ = 0.80, and with increasing Mondrian frequencies, *F*_(4, 44)_ = 16.40, *p* < 0.001, *N_P_*^2^ = 0.59, and we found a significant interaction parameter for these factors, *F*_(4, 44)_ = 5.68, *p* = 0.001, *N_P_*^2^ = 0.34. *Post-hoc* Bonferroni corrected comparisons showed that visibility for the two contrast values differed across all Mondrian frequencies except at the highest (28.5 Hz) frequency tested. The analysis of metacognition (Figure 3C) showed again an expected main effect of contrast, *F*_(1, 11)_ = 39.70, *p* < 0.001, *N_P_*^2^ = 0.78, and of Mondrian frequency, *F*_(4, 44)_ = 9.25, *p* < 0.001, *N_P_*^2^ = 0.45, on type II d prime, but no interaction parameter, *F*_(4, 44)_ = 1.47, *p* = 0.230. In conclusion, higher frequencies caused a consistent decrease in subjects' objective performance, subjective visibility ratings and metacognition.

### Experiment 3

From Experiment 2, it remains unclear whether the effect of the Mondrian frequencies on subjects' performance is due to the overall frequency of presentation of Mondrians or to the decreasing gap between the Mondrian that would place the targets temporally closer to their preceding and following Mondrians. We ran the following experiment in order to determine which of these two possibilities was causing the decreases in performance and the increases in suppression.

**Figure 4:**
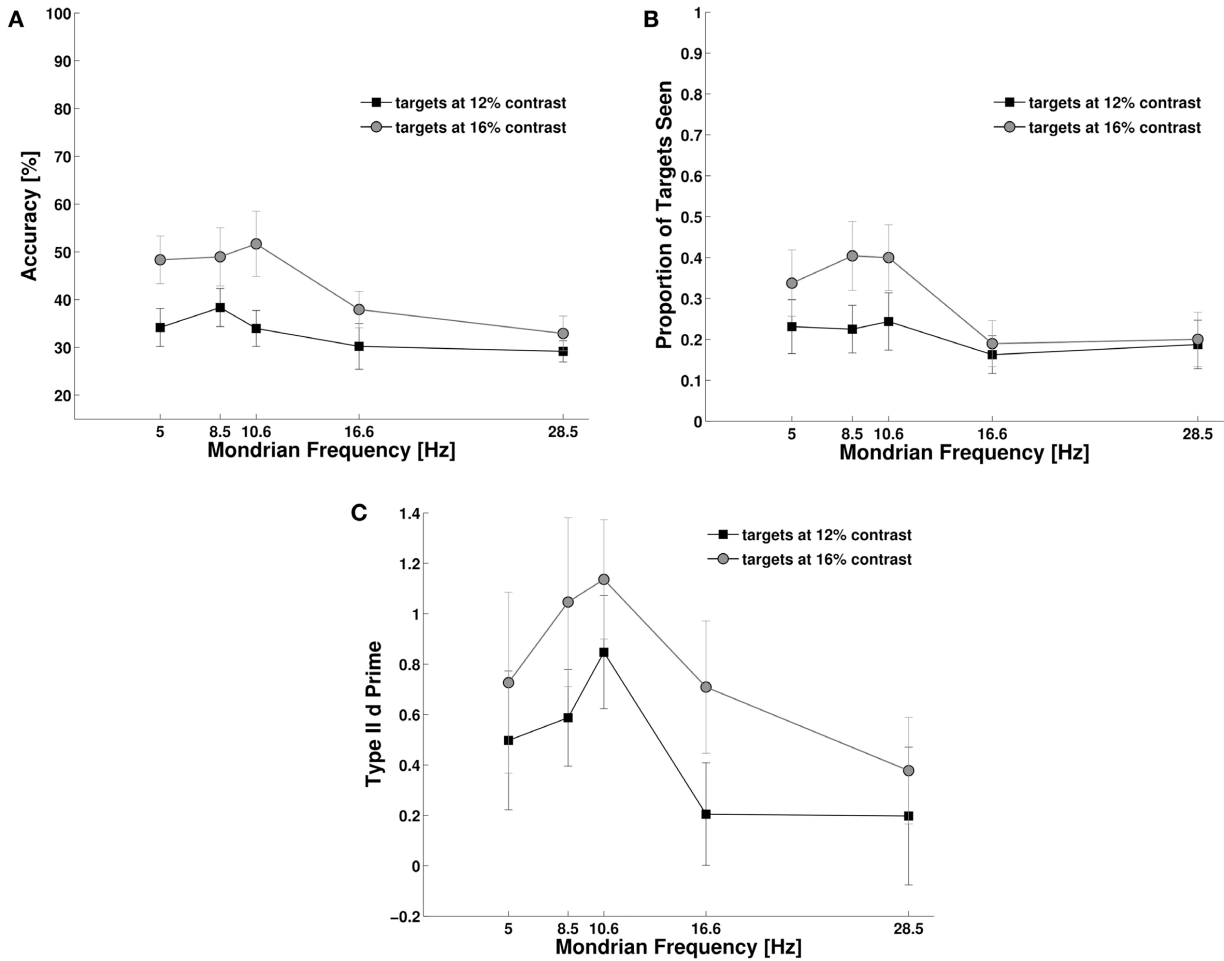
**Experiment 3**. Control experiment using only two Mondrian masks (“Sandwich Masking”). This experiment generated stronger target suppression than Experiment 2 (Figure 3, see Results Section for statistics), suggesting that the temporal proximity of the Mondrian with the target constitutes the main cause of suppression of transient targets. **(A)** Objective performance, **(B)** Subjective reports of visibility, and **(C)** Metacognition.

As shown in Figure 4, during dichoptic masking subjects' objective performance varied with Mondrian frequency, *F*_(4, 44)_ = 5.93, *p* = 0.03, *N_P_*^2^ = 0.35, and contrast, *F*_(1, 11)_ = 12.00, *p* = 0.005, *N_P_*^2^ = 0.52, (Figure 4A). We also observed an interaction between these main factors, *F*_(4, 44)_ = 2.71, *p* = 0.045, *N_P_*^2^ = 0.19. When we analyzed the effect of dichoptic masking on target visibility we found that both contrast, *F*_(1, 11)_ = 13.30, *p* = 0.003, *N_P_*^2^ = 0.54, and Mondrian frequency, *F*_(4, 44)_ = 5.37, *p* = 0.002, had an effect on target visibility, and that there was an interaction between the two factors, *F*_(4, 44)_ = 4.94, *p* = 0.004, *N_P_*^2^ = 0.31. On the other hand, the analysis of metacognition showed a marginal but non-significant effect of Mondrian frequency on type II d primes, *F*_(4, 44)_ = 2.48, *p* = 0.07, and no main effect for contrast, *F*_(1, 11)_ = 2.79, *p* = 0.123, nor for an interaction between the Mondrian frequency and contrast, *F*_(4, 44)_ = 0.68, *p* = 0.591.

Interestingly, inter-ocular “sandwich” masking was actually more effective than the CFS method used in Experiment 2 (Figure 3). Subjects' overall objective performance decreased during dichoptic masking in Experiment 3 compared with CFS in Experiment 2. Average performance was computed for each participant in Experiment 2 (12 participants) and Experiment 3 (12 participants) and the two groups were compared using a two-sample *t*-test: *t*_(22)_ = 3.31, *p* = 0.003, see Figures 3, 4.

### Experiment 4

**Figure 5:**
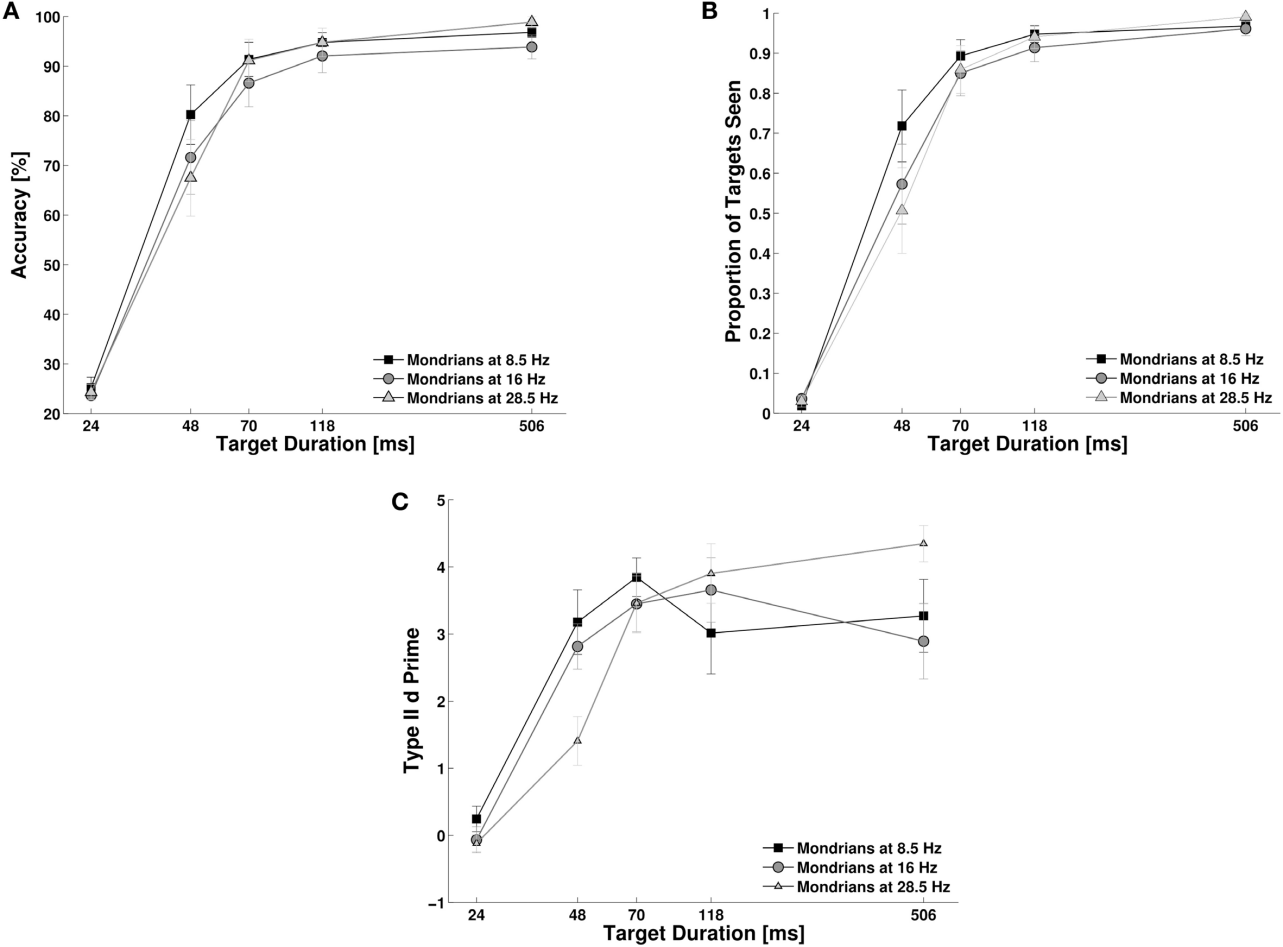
**Experiment 4**. Target duration modulated inter-ocular suppression during CFS and affected objective performance **(A)**, subjective reports of visibility **(B)**, and metacognition **(C)**.

We studied the effect of target duration and Mondrian frequency on objective accuracy, subjective reports and metacognition. First, we investigated subjects' objective performance for target detection. Objective performance moved from chance level to ceiling performance as target duration reached 70 ms (Figure 5A). A Two-Way repeated measures ANOVA, with target duration and Mondrian frequency as factors showed a main effect for target duration, *F*_(4, 40)_ = 1.6, *p* < 0.001, *N_P_*^2^ = 0.91, but only a marginal and statistically non-significant effect of Mondrian frequency, *F*_(2, 20)_ = 2.90, *p* = 0.078, *N_P_*^2^ = 0.22. However, we did observe a significant interaction parameter, *F*_(8, 80)_ = 3.84, *p* < 0.01, *N_P_*^2^ = 0.27. With the exception of the 48 ms condition, we could not observe any differences in the objective performance for each Mondrian frequency condition. For the 48 ms target duration condition we observed, in line with the results of Experiment 2, a better performance for lower frequencies than for higher masking frequencies (Figure 5A). This trend was larger between the 8.5 and the 28.5 Hz conditions (*t* = 2.53, *df* = 10, *p* = 0.029). Subjective reports of visibility (Figure 5B) also increased with target duration, *F*_(4, 40)_ = 93.20, *p* < 0.001, *N_P_*^2^ = 0.90, much in accordance with objective performance. Again, we did not observe a main effect of Mondrian Frequency, *F*_(2, 20)_ = 2.73, *p* = 0.089, *N_P_*^2^ = 0.21, but we found an interaction parameter between the factors, *F*_(8, 80)_ = 5.38, *p* < 0.001, *N_P_*^2^ = 0.35. As with objective performance, subjective reports were very low for the shortest target durations but they were at the ceiling of performance from 70 ms onwards. For the 48 ms condition, subjective reports were higher for the 8.5 Hz condition than for the 16 and 28 Hz condition. We observed a similar trend as with objective performance, in particular between the 8.5 and 28 Hz condition (*t* = 2.80, *df* = 10, *p* = 0.018). The analysis of metacognition (Figure 5C) showed again an expected main effect of target duration, *F*_(4, 40)_ = 38.30, *p* < 0.001, *N_P_*^2^ = 0.79, no effect of Mondrian frequency, *F*_(2, 20)_ = 0.162, *p* = 0.85, *N_P_*^2^ = 0.01 on type II d prime, but an interaction parameter, *F*_(8, 80)_ = 4.20, *p* = 0.001, *N_P_*^2^ = 0.29. Metacognition followed the same trend as objective performance and subjective reports, starting with a d prime of 0 for the 24 ms condition and reaching ceiling performance (d primes above 3) for the target durations of 70 ms or more. For the 48 ms condition, type II d primes were higher for the 8.5 and 16 Hz conditions than the 28 Hz condition (*t* = 2.37, *df* = 10, *p* = 0.039 and t = 2.40, *df* = 10, *p* = 0.036).

Overall, longer target durations caused a consistent increase in subjects' objective performance, subjective visibility ratings and metacognition. This increase showed a steep slope, subjects' performances went from chance level at 24 ms and reached near ceiling values at 70 ms target duration. Even though this modulation by target duration was irrespective of Mondrian frequency, at 48 ms target duration, we could observe that lower Mondrian frequencies generated better performance (confirming the results obtained in Experiment 2).

## Discussion

We showed that the temporal onset asynchrony between targets and Mondrians as well as the mask frequency both influence the strength of CFS for transient target stimuli. Smaller stimulus onset asynchronies (SOA) between Mondrians and targets generate stronger suppression (the forward and backward conditions in Experiment 1, see Figure 1A). Our results suggest that for transient targets CFS shows a trend of suppression similar to that reported in dichoptic visual masking (Macknik et al., 2000), flash suppression (Wolfe, 1984) and models of visual masking (Breitmeyer and Öğmen, 2006).

During CFS the depth of inter-ocular suppression varies depending on the spatial properties of the targets and Mondrians (Yang and Blake, 2012). We found that the temporal frequencies of Mondrians also influence the suppression of brief targets: higher temporal frequencies generated stronger suppression than lower frequencies. This modulation by Mondrian frequency occurred for CFS (Experiment 2) and for the brief presentation of only two Mondrians during “sandwich” masking (Experiment 3). Interestingly, “sandwich” masking resulted in the strongest suppression across all of the experiments, indicating that for brief target stimuli suppression was stronger for dichoptic masking than for CFS. These results, in addition to the fact that CFS constitutes the strongest technique for suppressing prolonged stimuli (Tsuchiya and Koch, 2005; Tsuchiya et al., 2006) suggest separate mechanisms of suppression operating for brief and prolonged stimuli (see below). An alternative interpretation of the finding that inter-ocular sandwich masking results in stronger suppression than CFS is that during CFS subjects might have established a temporal rhythm to facilitate the transient target detection. In our experiments we controlled for the effect of temporal attention on target detection by jittering target presentation time with regards to the onset time of Mondrians (which created a temporal uncertainty around target onset, see Materials and Methods Section). However, this jitter might not have been enough to fully discard the possibility of subjects establishing a temporal rhythm during the presentation of the Mondrians. Further studies are needed to clarify this point and to explain why, even if we consider plausible the hypothesis of an entrainment of attention, higher frequencies still generated stronger suppression of targets than lower frequencies.

Our results are consistent with a visual masking model that separates between a “sustained” and a “transient” channel for the detection of prolonged and transient stimuli (Breitmeyer and Öğmen, 2006). In this model brief targets are detected through the “transient channel” as triggered by the spatiotemporal edges of a stimulus (Macknik et al., 2000), while prolonged targets break into consciousness due to the “sustained channel” including more internally driven inhibition/adaptation and stochastic processes (Brascamp et al., 2006; Tsuchiya et al., 2006). The model starts from the anatomical distinction between Parvocellular (P) and Magnocellular (M) pathways in the retina toward lateral geniculate nucleus (LGN) and primary visual cortex. The two afferent streams are anatomically separated at the LGN level as well as at the input level of primary visual cortex (Lund, 1988; Callaway, 1998) and they show clear functional specialization (Croner and Kaplan, 1995). While the M pathway shows low contrast threshold, high luminance-contrast gain and short latency responses to visual stimuli (transient channel), the P pathway exhibits higher contrast thresholds, low contrast gain and sustained responses (Schmolesky et al., 1998). Crucially, the M pathway signals the appearance of stimuli and the rapid changes of location (motion) over time whereas the P pathway primarily signals pattern aspects such as the contours of stationary or slowly moving stimuli (Breitmeyer and Öğmen, 2006).

The model assumes that a mask that rapidly follows a visual stimulus interferes with the stimulus elaboration that would otherwise be performed by the transient channel, impeding visual stimuli detection. In this framework, CFS can be interpreted as a repeated resetting of the transient channel, resulting in decreased detection performance for brief visual stimuli presented together with the mask train (24–48 ms, see Figure 5). This is in line with the proposal that CFS does not constitute a stronger version of binocular rivalry, but a continuous repetition of flash suppression where each independent flash of Mondrian resets and renews inter-ocular suppression (Tsuchiya et al., 2006). It also suggests two types of masking mechanisms: a within-transient-channel masking for the suppression of transient targets with abrupt onsets and a between-transient-and-sustained channel masking for prolonged targets. Most previous studies using CFS have achieved long periods of invisibility of target stimuli (in the order of seconds) by adopting increasing ramps of contrast for the targets (Tsuchiya and Koch, 2005; Tsuchiya et al., 2006; Hesselmann and Malach, 2011; Stein and Sterzer, 2011; Stein et al., 2011). In our experiment 4, however, we show that targets with an abrupt onset break suppression with durations as short as 40 ms. We can explain the discrepancy between previous experiments with prolonged stimuli and our experiments with brief stimuli if we consider that the gradual ramp-up of the target contrast reduces the involvement of the transient channel. We hypothesize that since the transient channel plays an important role in the recruitment of the sustained channel, the latter does not get immediately activated. The train of masks leaves sustained targets presented with a ramp of contrast relatively unaltered, suggesting that in these cases the sustained channel (less affected by the Mondrians) performs target detection. Presumably, this yields long and stable suppression of the target stimuli. The sudden onset of counter-phase target stimuli in our experiments might have overcome the masking power of the CFS train at ~40 ms (Experiment 4), as would be expected given the higher sensitivity for flicker detection that characterizes the transient channel (Tolhurst, 1973).

Our findings show that the spatiotemporal dynamics of CFS affect stimuli visibility and cause differences among objective performance, subjective reports and metacognition. Previous research has shown that objective performance with near-threshold stimuli can be above chance level with little or no awareness of the presence of the target (Cheesman and Merikle, 1986; Kunimoto et al., 2001). In our experiments, visibility ratings remained in close agreement with objective discrimination accuracy. As shown in Experiment 1, subjects became more accurate and reported seeing the target more often for the “middle” condition compared to the “backward” condition. However, subjects' metacognition did not increase with visibility reports. While subjects became more accurate (objective sensitivity improved) and reported more targets as “seen,” they also made more errors with regard to their own judgments. This divergence between subjective and objective measures of visual awareness (see Figure 1) supports the idea that for stimuli presented near the threshold of visibility, metacognition often lags behind objective accuracy and subjective reports (Kunimoto et al., 2001).

In conclusion, our experiments extend the study of the interactions between inter-ocular masking and CFS. They provide a better understanding of the dynamics of CFS with transient onset stimuli and of the robust similarities between transient target CFS and dichoptic visual masking. Changes in CFS dynamics that render stimuli more or less visible may cause differences among objective performance, subjective reports and metacognition. As our study shows, generalization is often not easy to achieve since a number of related mechanisms of visual perception can summate in different ways for each specific masking technique. For this reason, we stress the need for a careful consideration of the type of psychophysical techniques employed, the way subjects' responses are interpreted, and the type of stimuli being rendered invisible before comparing results among studies and inferring non-conscious processes.

### Conflict of interest statement

The authors declare that the research was conducted in the absence of any commercial or financial relationships that could be construed as a potential conflict of interest.

## Supplementary material

Supplementary Figure 1 **We compared objective performance, visibility and metacognition within Experiment 1**. The different measures were normalized by computing the z-score of each dependent variable across all conditions and participants. Using this normalized measure we computed the difference between “middle mask condition” and “backward condition” for each participant and dependent variable. The average difference is reported across the 3 dependent variables (see x axis labels). The difference between “middle mask condition” and “backward condition” obtained with normalized objective performance is significantly different than with normalized metacognition (*p* < 0.05). Moreover, the difference between “middle mask condition” and “backward condition” obtained with normalized visibility is significantly different than with normalized metacognition (*p* < 0.05, see main text for details).
