# Supplementary figures and images for: Waves of visibility: probing the depth of inter-ocular suppression with transient and sustained targets
Source: Front Psychol. 2014 Jul 30;5:804. doi: 10.3389/fpsyg.2014.00804 (PMC4115669; doi:10.3389/fpsyg.2014.00804)

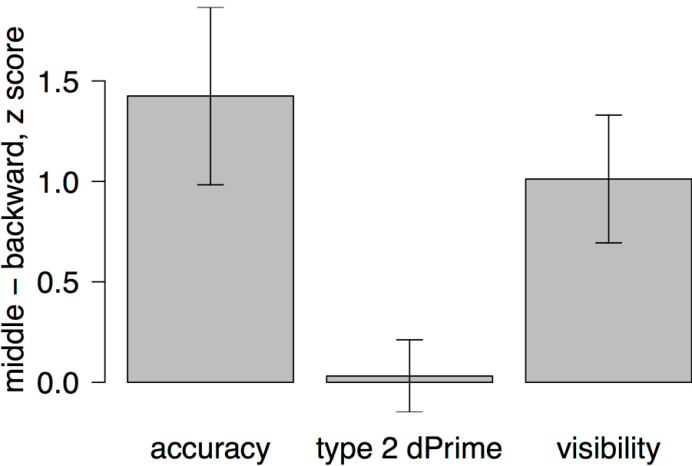

Supplement: Supplementary Figure 1 — We compared objective performance, visibility and metacognition within Experiment 1. The different measures were normalized by computing the z-score of each dependent variable across all conditions and participants. Using this normalized measure we computed the difference between “middle mask condition” and “backward condition” for each participant and dependent variable. The average difference is reported across the 3 dependent variables (see x axis labels). The difference between “middle mask condition” and “backward condition” obtained with normalized objective performance is significantly different than with normalized metacognition (p < 0.05). Moreover, the difference between “middle mask condition” and “backward condition” obtained with normalized visibility is significantly different than with normalized metacognition (p < 0.05, see main text for details). [file Presentation1.PDF]
